# Missing call bias in high-throughput genotyping

**DOI:** 10.1186/1471-2164-10-106

**Published:** 2009-03-13

**Authors:** Wenqing Fu, Yi Wang, Ying Wang, Rui Li, Rong Lin, Li Jin

**Affiliations:** 1 MOE Key Laboratory of Contemporary Anthropology and Center for Evolutionary Biology, School of Life Sciences and Institutes of Biomedical Sciences, Fudan University, Shanghai 200433, PR China; 2 Chinese National Human Genome Center at Shanghai, Shanghai 201203, PR China; 3 CAS-MPG Partner Institute for Computational Biology, Shanghai Institutes for Biological Sciences, Chinese Academy of Sciences, Shanghai 200031, PR China

## Abstract

**Background:**

The advent of high-throughput and cost-effective genotyping platforms made genome-wide association (GWA) studies a reality. While the primary focus has been invested upon the improvement of reducing genotyping error, the problems associated with missing calls are largely overlooked.

**Results:**

To probe into the effect of missing calls on GWAs, we demonstrated experimentally the prevalence and severity of the problem of missing call bias (MCB) in four genotyping technologies (Affymetrix 500 K SNP array, SNPstream, TaqMan, and Illumina Beadlab). Subsequently, we showed theoretically that MCB leads to biased conclusions in the subsequent analyses, including estimation of allele/genotype frequencies, the measurement of HWE and association tests under various modes of inheritance relationships. We showed that MCB usually leads to power loss in association tests, and such power change is greater than what could be achieved by equivalent reduction of sample size unbiasedly. We also compared the bias in allele frequency estimation and in association tests introduced by MCB with those by genotyping errors. Our results illustrated that in most cases, the bias can be greatly reduced by increasing the call-rate at the cost of genotyping error rate.

**Conclusion:**

The commonly used 'no-call' procedure for the observations of borderline quality should be modified. If the objective is to minimize the bias, the cut-off for call-rate and that for genotyping error rate should be properly coupled in GWA. We suggested that the ongoing QC cut-off for call-rate should be increased, while the cut-off for genotyping error rate can be reduced properly.

## Background

Driven by the common disease-common variant (CDCV) hypothesis [1], genome-wide association (GWA) studies have demonstrated its power in the identification of genetic variants underlying the diseases [2-5]. The completion of the human genome sequence [6,7] and the International HapMap Project [8-10] as well as the advent of highly efficient and affordable genotyping technologies made GWA within reach. The Phase II HapMap contains more than 4.3 million common SNPs and the coverage is estimated to capture 94% of common variation in CEU and CHB+JPT and 81% in YRI with *r*^2 ^≥ 0.8[9]. Several high-throughput and cost-effective technologies for genotyping that are currently being used, they are TaqMan assay [11] and GeneChip array [12] (based on hybridization with allele-specific probes), SNPstream system [13] and GoldenGate assay [14] (based on single nucleotide primer extension), Invader assay [15] (based on enzymatic cleavage), and SNiPer [16] (based on Oligonucleotide ligation). Though different reaction mechanisms are employed in different methods, fluorescence detection is widely employed in the process of the specific allele detection.

To deal with abundant genotype data produced by various genotyping platforms, quality control (QC) to ensure the accuracy of allele call becomes a critical issue. When genotyping errors occur, its effects on linkage analysis [17-19], LD measures [20,21], tagging SNP selection [22] and the subsequent association tests [22-24] have been widely and carefully investigated. Various strategies of detecting genotyping errors or removing its effects on analyses, especially on linkage analysis have been proposed [25-28]. In addition to genotyping errors, missing calls seem to be abundant in high-throughput genotyping. For example, in the data of the Phase I HapMap, less than 20% data that failed to pass QC was due to genotyping error (>1 duplicate inconsistent or >1 medelian error), while more than 65% of the markers show missing data in over 20% individuals [9]. The presence of missing calls was even more prominent in the Phase II data of HapMap [8]. However, the effect of missing call on the subsequent analyses has been largely ignored.

Strong emphasis on the accuracy of allele calls and technical success to achieve that has made the effect of missing call largely overlooked. It was suggested that 'no-call' procedure should be taken where observations of borderline quality be removed from allele calls in order to keep the genotyping error rate as low as possible [29]. This 'no-call' principle becomes a common practice in genotyping procedures. However, it should be noted that the validity of the 'no-call' principle relies on an implicit assumption that genotyping frequencies in no-call individuals are equal to those in the population. Under this hypothesis, missing data from the no-call procedure simply leads to a power loss due to a decreased sample size, and does not affect the estimation of allele frequencies at all. In this report, we started with a close examination of the validity of the 'no-call' principle by regenotyping those individuals whose genotypes that cannot be unequivocally determined and therefore would have been otherwise discarded. The objectives of this report are (1) to demonstrate experimentally how widely and seriously the problem of missing call bias (MCB) exists, (2) to investigate theoretically the effects of MCB on the subsequent analyses, especially on association studies, and (3) to provide suggestion on dealing with observations of borderline quality and re-evaluate the current QC standards, through comparing the effects of MCB and genotyping errors on allele frequency estimation and association studies.

## Results

There are two major causes for missing calls. One is due to poor quality of DNA samples, which often fails to be amplified and to generate strong enough intensity of fluorescence signals over the background. The other arises when an observation, i.e., a read out of fluorescence signals, cannot be assigned unequivocally to any of the clusters of genotype, therefore, is subject to 'no-call' procedure. In this report, we mainly focus on the missing calls due to the failure of being assigned to any clusters of genotype.

### Nature of no-calls: results of sequencing

To evaluate the nature of no-calls in reality, four different widely-used high-throughput genotyping platforms were included in this study, and they are GenomeLab™ SNPstream Genotyping System (Beckman Coulter, Los Angeles), BeadLab SNP Genotyping System (Illumina, San Diego), TaqMan^® ^SNP Genotyping Assays (ABI, Foster City) and GeneChip^® ^Human Mapping 500 K Array Set (Affymetrix, Santa Clara). Eight SNPs were selected and subjected to regenotyping of equivocal observations (no-calls) through sequencing. The criteria for the selection of SNPs and samples for sequencing were presented in Methods.

The genotype distribution of the *observed *data at each locus which were produced by the respective genotyping technology was compared with that of no-calls which were obtained by sequencing (Table 1). Statistically significant differences were observed in SNPstream, Illumina and GeneChip 500 K, indicating that the MCB indeed exists in widely-used genotyping technologies and it would lead to a biased estimation of allele/genotype frequencies. The genotype-specific call-rates *c*_*i*_(*i *= *AA*, *Aa*, *aa*) were calculated, most of which were above 0.95, but it could be as low as 0.75 for GeneChip 500 K.

**Table 1:** The regenotyping results for the different genotyping platforms.

**Platform**	**rsID**		***Obs***.	***Seq***.	***c*_*i*_**	**pVal**
SNPstream	*rs6743724*	*AA*	47	0	1.000	0.231
		*Aa*	427	2	0.995	
		*aa*	1009	16	0.984	
	
	*rs699512*	*AA*	239	3	0.987	**0.0277**
		*Aa*	1064	3	0.997	
		*aa*	1309	15	0.989	

Illumina	*rs2277632*	*AA*	351	8	0.978	**1.42 × 10^-5^**
		*Aa*	1129	1	0.999	
		*aa*	941	2	0.998	
	
	*rs1457043*	*AA*	449	1	0.998	0.463
		*Aa*	1198	6	0.995	
		*aa*	775	1	0.999	

TaqMan	*rs10109984*	*AA*	111	1	0.991	0.0898
		*Aa*	480	9	0.981	
		*aa*	513	20	0.962	
	
	*rs11226*	*AA*	242	4	0.984	0.599
		*Aa*	568	17	0.971	
		*aa*	298	8	0.974	

GeneChip 500 K	*rs11928855*	*AA*	66	22	0.750	**2.26 × 10^-15^**
		*Aa*	243	1	0.996	
		*aa*	133	1	0.992	
	
	*rs6855202*	*AA*	59	15	0.797	**3.33 × 10^-13^**
		*Aa*	246	0	1.000	
		*aa*	141	0	1.000	

pVal is the *P *value calculated by Fisher Exact Test to show whether the genotype counts called by the software provided by the respective platform (*Obs*.) were significantly different from the no-calls' counts by sequencing (*Seq*.). *c*_*i *_is the genotypic specific call-rate for *AA*, *Aa *and *aa*.

**Figure 1:**
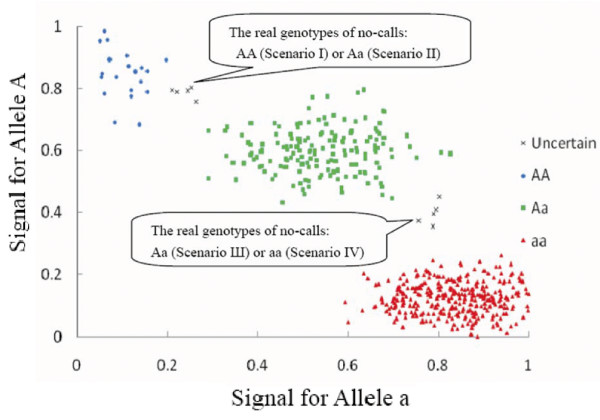
**The sketch of genotyping calling**. The points shown in 'x' represent no-calls due to the failure of being assigned to any clusters of genotype unequivocally. When the data points lie between the cluster of homozygotes of minor alleles (*AA*) and that of heterozygotes (*Aa*), the real calls could be *AA *or *Aa*, corresponding to Scenario I and Scenario II. When the data points lie between the cluster of heterozygotes (*Aa*) and that of homozygotes of major alleles (*aa*), the real calls could be *Aa *or *aa*, corresponding to Scenario III and Scenario IV.

In the subsequent sections, in order to explore the effects introduced by MCB, we proposed a model to investigate the nature of no-calls. Typically an equivocal observation occurs as follows (Fig. 1). For the data points that lie between the cluster of homozygotes of minor alleles (AA) and the cluster of heterozygotes (Aa), the real genotypes could be either homozygotes of minor alleles (Scenario I) or heterozygotes (Scenario II). For those that lie between the cluster of homozygotes of major alleles (aa) and the cluster of heterozygotes (Aa), the real genotypes could be either heterozygotes (Scenario III) or homozygotes of major alleles (Scenario IV). When the observations that cannot be called unequivocally are discarded, Scenario II is equivalent to Scenario III. To facilitate discussion, we assumed no-calls only happen in a specific genotype with the genotype-specific call-rate *c *(0 ≤ *c *≤ 1).

### Effect of MCB on type-I error rate for HWE

Hardy-Weinberg Equilibrium has been repeatedly recommended as a measure for QC in the context of genetic association studies [30]. In the following, we will show that MCB is one of causes for the departure from HWE.

**Figure 2:**
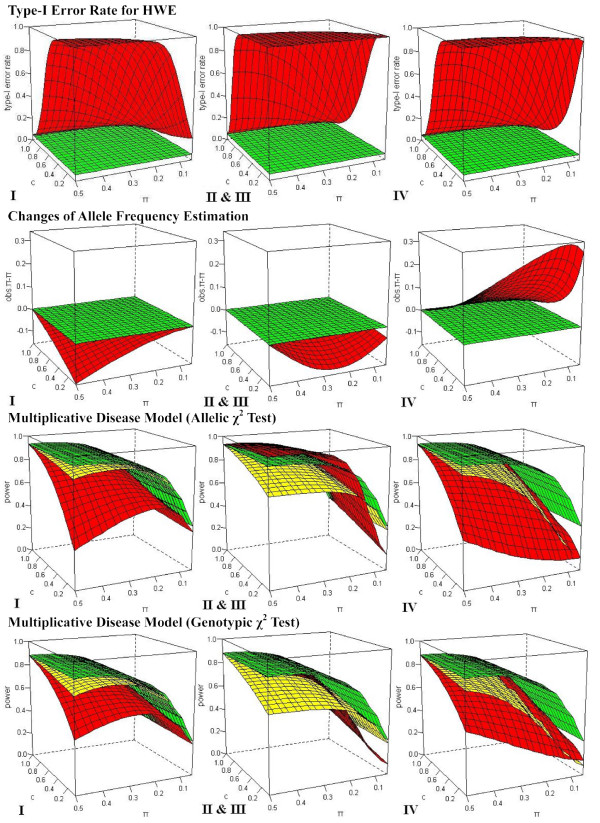
**Effects of MCB on HWE, MAF estimation and association studies under multiplicative disease model**. The comparison was conducted among the null (printed in green), UBM (printed in yellow) and MCB (printed in red). The figures correspond to Scenario I, Scenario II & III and Scenario IV from the left to right with different extents of missing bias (0 ≤ *c *≤ 1).

The type-I error rate for departure from HWE disturbed by MCB is inflated with the increasing of *c *(Fig. 2). When MCB happens in homozygotes (Scenario I and Scenario IV), it leads to a departure from HWE because of excessive heterozygotes. The inflation is similar for *AA *and *aa *for a given *c*. When MCB happens in heterozygotes (Scenario II & III), excessive homozygotes result in the departure from HWE. The inflation is more pronounced for MCB in heterozygotes than that in homozygotes. For example, the type-I error rate is 0.055 ~0.228 in Scenario I and Scenario IV, while it can be 0.076 ~0.654 in Scenario II & III under different MAFs in the presence of MCB (*c *= 0.80) for a population (*N *= 500) under HWE in the significant level of 0.05. However, HWE still holds, as expected, when missing equivalently but unbiasedly across different genotypes (Unbiased Missing, UBM).

### Effect of MCB on allele frequency estimation

The accuracy of allele/genotype frequency estimation is of special importance since many analyses such as association studies, inference of haplotype, and inference of population structure rely on it. UBM does not affect allele/genotype frequency estimation although it reduces the sample size. However, MCB does. When missing bias in Scenario I and Scenario II & III, MAF is underestimated; and when in Scenario IV, MAF is overestimated and this change is larger than the former two (Fig. 2). It should be noticed that the change of allele frequency in Scenario IV can be quite serious, especially for the locus with rare MAF. For instance, the change of MAF can be 0.019 with *c *= 0.80, 0.068 with *c *= 0.50, and even to 0.184 with *c *= 0.20, while the real value of MAF is only 0.10.

### Effect of MCB on association studies

The development of high-throughput genotyping technologies make association study widely conducted for identification of disease loci underlying complex traits. We now examine the effect of MCB on association using various disease models and statistical tests.

Power issue is of special importance in association studies [31,32]. To investigate the effect of MCB on the power of association studies, MCB was introduced into disease models (see Methods). Here, the sample size is 500 for both case and control groups, and MCB are identical for both groups.

It has been commonly assumed that missing calls would lead to power loss due to decreased sample size, which only holds in absence of MCB. In the presence of MCB, the power can be affected by both the sample size and the biased estimation of allele and genotype frequencies (see Fig. 2, Additional file 1 &2). The change of power by MCB is usually larger than that by UBM. For example, the power loss by UBM is all less than 5% for the locus with MAF = 0.25 under various disease models (power ≈ 80% in genotypic *χ*^2 ^test) when *c *= 0.80, while the change can be around and even more than 10% when disturbed by MCB (Table 2).

**Table 2:** The power under different modes of inheritance relationship in the significant of 0.05.

**Disease Models**	**Penetrances (*h*_*AA*_, *h*_*Aa*_, *h*_*aa*_)**	**Scenarios**	**Power in Allelic *χ *^2^test**			**Power in Genotypic *χ *^2^test**		
			
			Null	MCB	UBM	Null	MCB	UBM
		I	0.805	0.790	0.800	0.806	0.800	0.800
Dominant	(0.0148, 0.0148, 0.01)	II & III	0.805	0.799	0.770	0.806	0.762	0.769
		IV	0.805	0.730	0.762	0.806	0.756	0.759

		I	0.619	0.472	0.611	0.801	0.710	0.794
Recessive	(0.0201, 0.01, 0.01)	II & III	0.619	0.691	0.586	0.801	0.798	0.769
		IV	0.619	0.599	0.569	0.801	0.796	0.751

		I	0.495	0.536	0.490	0.796	0.792	0.791
Overdominant	(0.01, 0.0148, 0.01)	II & III	0.495	0.447	0.461	0.796	0.736	0.758
		IV	0.495	0.406	0.453	0.796	0.757	0.748

		I	0.869	0.831	0.864	0.800	0.770	0.793
Additive	(0.0178, 0.0139, 0.01)	II & III	0.869	0.880	0.840	0.800	0.776	0.763
		IV	0.869	0.816	0.831	0.800	0.756	0.752

		I	0.875	0.831	0.870	0.800	0.763	0.793
Multiplicative	(0.01847, 0.01359, 0.01)	II & III	0.875	0.889	0.847	0.800	0.782	0.763
		IV	0.875	0.826	0.838	0.800	0.759	0.752

MAF is 0.25 and the sample size in case and control is 500 respectively. Null, the power in absence of missing; MCB, the power affected by missing call bias; UBM, the power when missing equivalently but unbiasedly across different genotypes.

For the *χ*^2 ^test based on genotype frequencies, MCB always leads to power loss in all scenarios under different disease models compared with the null (in the absence of missing) (see Fig. 2 and Additional file 2). But for the *χ*^2 ^test based on allele frequencies, it even can gain the power in some scenarios, because of the biased estimation of allele frequency (see Fig. 2 and Additional file 1). Genotypic *χ*^2 ^test seems to be more robust to the changes of power in association studies than allelic *χ*^2 ^test in the presence of MCB (see Fig. 2, Additional file 1 &2, and Table 2).

The changes of power vary in different disease models. It can be summarized that for the disease model with dominant relationship (*h*_*AA *_= *h*_*Aa *_≠ *h*_*aa*_), the power change in Scenario IV (missing in *aa*) is most; for the disease model with recessive relationship (*h*_*AA *_≠ *h*_*Aa *_= *h*_*aa*_), the power change in Scenario I (missing in *AA*) is the largest; for the disease model with overdominant relationship (*h*_*Aa *_≠ *h*_*AA *_= *h*_*aa*_), the power change in Scenario II & III (missing in *Aa*) is the most; and for the disease model with additive relationship (*h*_*AA*_>*h*_*Aa*_>*h*_*aa*_), the power change in Scenario I is similar to that in Scenario IV, especially when *c *is small, while the decrease of sample size in quantity differs greatly (see Additional file 1 &2). The influence under the disease model with multiplicative relationship (*h*_*AA*_>*h*_*Aa*_>*h*_*aa*_) is similar to that with additive relationship (Fig. 2).

In addition, though the minor allele of *A *in the current settings of disease models is susceptible to the disease (in overdominant disease model, *Aa *is susceptible to disease), the conclusions drawn above can also be extended when A is a protective one (data not shown). Moreover, in the disease model (*h*_*AA *_= *0.01*, *h*_*Aa *_= *0.01, and h*_*aa *_= *0.01*), the type-I error rate in MCB remains to be 0.05, indicating that MCB does not inflate the false positive rate for the association studies, under the assumption that the extent of missing is identical in both case and control.

### Tradeoff between MCB and genotyping errors

In the previous section, we showed that MCB is common in the current genotyping technologies, and it could affect the subsequent analyses seriously and lead to false conclusions. The key issue is how to deal with those equivocal observations which apparently are responsible for MCB. Two alternative options are available. The first option is to discard the observations of borderline quality using the 'no-call' procedure which may lead to MCB. The second option is to assign these observations to one of the genotypes at the cost of increasing genotyping errors. Here, we compare the overall outcome (allele frequency estimation and power of association studies) of these two options and try to offer guidelines for different scenarios to minimize the biases caused by the equivocal observations. In addition, we evaluate the overall call-rate and genotyping error rate in the two options respectively and intend to re-examine the current QC standards.

To facilitate the presentation, the genotype-specific call-rate *c *was set to 0.80, a moderate MCB as shown previously. For the second option, we assumed all of the equivocal observations are called and the proportion of accurate calls among these equivocal ones is denoted by *conf *(0 ≤ *conf *≤ 1). The genotyping error rate increases with decreasing *conf*. For instance, if *conf *= 1, it means all of the equivocal observations are called accurately; whereas, if *conf *= 0, all of the equivocal ones are misclassified.

**Figure 3:**
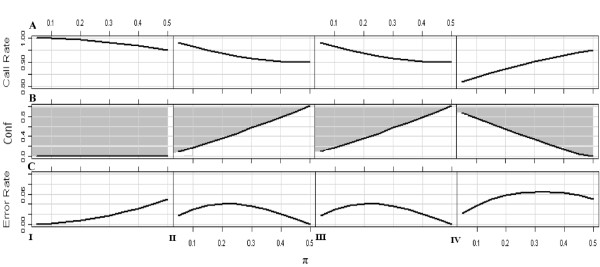
**Effects of MCB and genotyping errors on MAF estimation**. **A) **illustrates the overall call-rate for the loci with different MAFs in the presence of MCB (*c *= 0.8). **B) **illustrates the threshold of *conf *by a solid line. If the equivocal observations can be called accurately in a confidence above the *conf *threshold, it prefers to call those equivocal ones at the cost of genotyping errors to minimize the biased estimation of MAF introduced by the equivocal observations (grey area above the line). Otherwise, 'no-call' procedure is beneficial, which results in MCB (area below the line). **C) **illustrates the genotyping error rate, when the equivocal observations are called in the *conf *threshold mentioned above. The figures correspond to Scenario I, Scenario II, Scenario III and Scenario IV from the left to right.

Both MCB and genotyping errors will possibly lead to inaccurate estimation of allele/genotype frequencies and in turn distorted association. When 'no-call' procedure is applied to those observations of borderline quality, we showed earlier that the biased estimation is dictated only by MAF and *c*. When the equivocal observations are called, the bias in allele frequency estimation depends on *conf *in addition to MAF and *c*. In particular, the bias in allele frequency estimation reflected by the changes of MAF estimation increases with the decreasing *conf *(see Additional file 3). Fixed MAF and *c*, the biased estimations caused by MCB and by genotyping errors are comparable. The bias introduced by MCB is certain, whereas the bias caused by genotyping errors changes with the *conf *monotonically. Interestingly, when the *conf *is large enough (indicated by the solid line in Fig. 3B), the biased estimation of MAF caused by genotyping errors are smaller than that caused by MCB. Therefore, it would be more beneficial to call the equivocal observations in this case (grey area above the line in Fig. 3B). However, when the *conf *is below the solid line indicated in Fig. 3B, the biased estimation of MAF caused by genotyping errors is more and 'no-call' procedure is recommended (area below the line in Fig. 3B). It should be noted that the bias in estimation of allele frequency in Scenario I by MCB is the greatest, more so than in genotyping errors, even with the highest error rate (*conf *= 0). Therefore, it is suggested that 'no-call' principle should not be taken in Scenario I if the objective is to minimize the biased estimation of MAF (Fig. 3).

**Figure 4:**
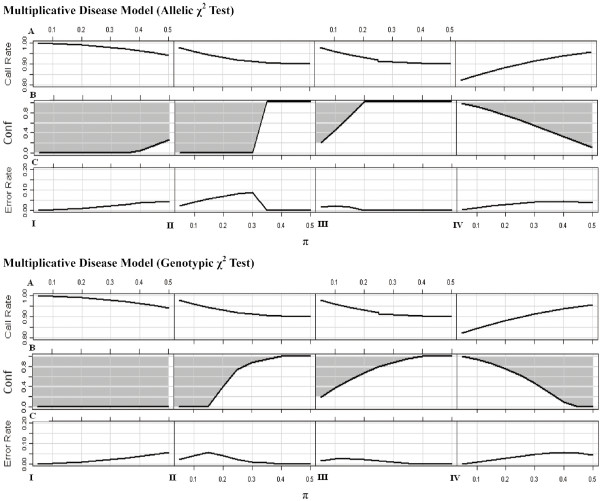
**Effects of MCB and genotyping errors on association studies under multiplicative disease model**. **A) **illustrates the overall call-rate for the loci with different MAFs in the presence of MCB (*c *= 0.8). **B) **illustrates the threshold of *conf *by a solid line. If the equivocal observations can be called accurately in a confidence above the *conf *threshold, it prefers to call those equivocal ones at the cost of genotyping errors to minimize the power loss (grey area above the line). Otherwise, 'no-call' procedure is beneficial, which results in MCB (area below the line). **C) **illustrates the genotyping error rate, when the equivocal observations are called in the *conf *threshold mentioned above. The figures correspond to Scenario I, Scenario II, Scenario III and Scenario IV from the left to right.

In the following section, we explore the performance of association studies affected by MCB and by genotyping errors. Here, MCB (*c *= 0.80) and genotyping errors (*c *= 0.80, 0 ≤ *conf *≤ 1), are assumed to be identical for case and control groups. MCB and genotyping errors were introduced to the disease models with various modes of inheritance relationship (Table 2) respectively according to Methods. The power affected by MCB was discussed previously. The power affected by genotyping errors were shown in Additional file 3. For *χ*^2 ^test based on genotype frequencies, genotyping errors may have no effect on association studies sometimes, *i.e*., in Scenario I and Scenario II for the dominant disease model (*h*_*AA *_= *h*_*Aa *_≠ *h*_*aa*_); otherwise, it may cause power loss. But for *χ*^2 ^test based on allele frequencies, it may occasionally cause power gain because of the biased estimation of allele frequency. Though the power affected by genotyping errors is complicated in different scenarios and disease models, the power either does not change or changes monotonically with the *conf *given the scenarios and disease models (see Additional file 3). Therefore, similar to the previous study on allele frequency estimation, a threshold of *conf *(indicated by the solid lines in Fig. 4B, and Additional file 4B &5B) is expected as well. If the *conf *is below the threshold, the power loss caused by genotyping errors is larger than that by MCB; therefore, 'no-call' procedure should be taken (area below the line in Fig. 4B, and Additional file 4B &5B). Otherwise, it is better to call the observations of borderline quality at the cost of genotyping errors (grey area above the line in Fig. 4B, and Additional file 4B &5B). For instance, for a locus with MAF = 0.35, when 'no-call' procedure is taken for the equivocal observations happened in Scenario I (*c *= 0.80), the overall call-rate can still achieve at 97.6%. The power is 87.7% in MCB compared with 92.1% of the null in the multiplicative disease model for allelic *χ*^2 ^test. However, if these equivocal observations are called even though they are completely misclassified (*conf *= 0), the power with genotyping errors can be 88.1% at least. It indicates that in order to reduce the power loss caused by the equivocal observations, it would be more beneficial to call the equivocal observations with a genotyping error rate 2.5% than 'no-call' with an overall call-rate 97.5% (Fig. 4).

As shown above, the commonly-used 'no-call' principal for the observations of borderline quality is not always the best choice. By weighing the influences on the performance of association study and allele frequency estimation, it is therefore preferable to force the calling of, even though they can be erroneous, these equivocal observations when they lie between the cluster of homozygotes of minor alleles and that of heterozygotes (i.e. in Scenario I & Scenario II). When the equivocal observations lie between the cluster of homozygotes of major alleles and the cluster of heterozygotes (i.e., in Scenario III & Scenario IV), the loss of power introduced by MCB is more pronounced than that by genotyping error when these equivocal observations can be accurately called; but when the calling accuracy cannot be granted (the *conf *is small), the power loss is affected more by the genotyping error and it may be better to invoke 'no-call' procedure. In addition, with different disease models, different decisions for dealing with these equivocal observations may be made. A program called *QC-Tradeoff *is available online to suggest whether 'no-call' procedure could be conducted  to minimize the biases caused by the equivocal observations.

**Figure 5:**
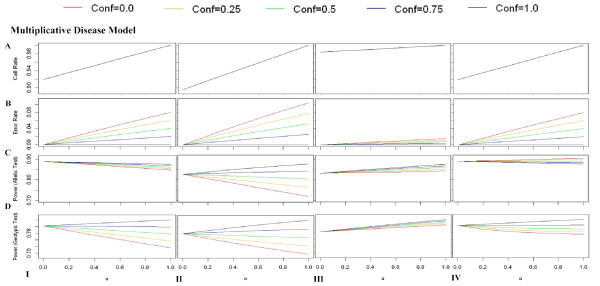
**Joint effects of MCB and genotyping errors on association studies under multiplicative disease model**. **A) **illustrates the overall call-rate for the loci with different values of *α *in the joint models of MCB and genotyping errors (MAF = 0.25, *c *= 0.8, *conf *= 0.0, 0.25, 0.5, 0.75 and 1.0). **B) **illustrates the genotyping error rate with different values of *α *in the corresponding joint models. **C) **illustrates the power based on allelic *χ*^2 ^test with different values of *α*. **D) **illustrates the power based on genotypic *χ*^2 ^test with different values of *α*. The figures correspond to Scenario I, Scenario II, Scenario III and Scenario IV from the left to right.

In the above analyses, for the models of genotyping errors, we assumed all of equivocal observations were called to facilitate the discussion. Here, we extended a general model to explore the joint effects caused by MCB and genotyping errors. We assumed only (100 × *α*)% of equivocal observations (0 ≤ *α *≤ 1) were called with accuracy still denoted by *conf*. The genotype frequencies in this joint model were illustrated in Additional file 7. When *α *= 0, this model is equivalent to the model of MCB; and when *α *= 1, this model is equivalent to the model of genotyping errors. We introduced this joint model (*c *= 0.8, *conf *= 0.0, 0.25, 0.5, 0.75. 1.0, and 0 ≤ *α *≤ 1) to the disease models denoted in Table 2. Fig. 5 and Additional file 6 illustrated the power of association tests in the presence of MCB and genotyping errors, and the corresponding overall call-rate and genotyping error rate. An interesting finding is that the influences of the power caused by the equivocal observations always change monotonically with *α *from 0 (the model of MCB) to 1(the model of genotyping error). It indicates in order to minimize the biases caused by the equivocal observations on association studies, the validate procedure is either no-call resulted in MCB or call all of the equivocal observations with genotyping errors, which we had discussed above.

Given the knowledge of relationship of bias in allele/genotype frequency estimation and in association study with the magnitude of MCB and genotyping errors, it is therefore likely to develop a strategy to minimize the bias by choosing proper cut-offs for call-rate and genotyping error rate (see Discussion).

## Disccusion

The advent of high-throughput genotyping technologies led to an exciting era of genome-wide associations. Genotype data with good quality are imperative in ensuring the creditability of a study. Missing calls in high-throughput genotyping has long been ignored in genetic studies. In this study, we demonstrated experimentally the prevalence and severity of the problem of missing calls, especially MCB, in the current genotyping technologies.

We also showed theoretically how MCB could lead to biased conclusions in the subsequent analyses including estimation of allele/genotype frequencies and association tests. MCB leads to power loss in most cases, and such loss may lead to false negative conclusions. Compared with allelic *χ*^2^test, genotypic *χ*^2 ^test is more robust to MCB. Various modes of inheritance relationship (dominant, recessive, overdominant, additive and multiplicative) were considered in our study. We also showed that when missing bias happens in the genotype whose contribution to the disease differs most, regardless whether it is susceptible or protective to the disease, it affects the power of association studies most.

In this study, we investigated the bias of association in the presence of both MCB and genotyping errors, and demonstrated that they contributed to the bias differently. This result is of special importance in determining the cut-offs used for QC in the current practice of GWA. The question is whether the current QC standards are optimal. If the objective is to minimize the bias in allele/genotype frequency estimation and in association tests, the cut-off for call-rate and that for genotyping error rate should be properly coupled in GWA. This leads to a re-examination of the existing QC standards for both call rate and genotyping error rate that are widely used in various association studies.

A commonly used QC standard for call rate is 80% and 95% or above for the first screening and fine mapping, respectively, in GWAs (e.g. Easton et al. [3]; Hunter et al. [4]). Although we demonstrated that the bias in allele frequency estimation and in association study discussed in Results (Fig. 3 &4, and Additional file 4 &5) is not negligible when 'no-call' procedure is applied, their call-rates are all above 80% and even can be above 95%. It suggests that the existing cut-offs are not sufficiently stringent to filter out the loci which may suffer from MCB.

A genotyping error rate < 1% is considered acceptable [3-5,33]. This is an extremely stringent cut-off in the presence of equivocal observations, given that such a stringent cut-off would force to invoke 'no-call' principal whereas would not lead to a reduction of bias. Our results indicated that in most cases, the bias can be greatly reduced by increasing the call-rate at the cost of genotyping error rate, i.e., < 5% (Fig. 3 &4, and Additional file 4 &5). Therefore, we suggested that the ongoing QC cut-off for call-rate should be increased, while the cut-off for genotyping error rate can be reduced properly.

A program called *QC-Tradeoff *is available online to provide a *conf *threshold. If the threshold is high, it is conservative to take 'no-call' procedure to reduce the power loss in association studies introduced by the equivocal observations; otherwise, the equivocal ones could be called even though genotyping errors may be occurred. Moreover, we showed that the missing calls can usually be reduced sufficiently but with certain accuracy using the current technologies, indicating that the value of *conf *in reality is usually high enough. Through adjusting relevant parameters which are implemented in the calling software provided by genotyping platforms (such as Illumina, TaqMan and GeneChip 500 K), it allows either higher call rates or greater genotyping rate. For example, we adjusted quality value of TaqMan from 0.95 (default) to 0.80 to illustrate the change of calling in the two loci *rs10109984 *and *rs11226*. After a change of the quality value, the overall call-rate increased substantially. In particular, when the quality value is 0.95, 30 and 29 calls were not called for the loci *rs10109984 *and *rs11226*, respectively. When 0.8 was chosen as the quality value, only 5 were not called at *rs10109984 *and 7 at *rs11226*. Subsequently, the number of genotyping discordance between the genotyping results and sequencing results was 8 (corresponding to *conf *= *0.73*) and 0 (corresponding to *conf *= *1.0*) for the locus *rs10109984 *and *rs11226*, respectively.

Furthermore, our results also suggested that MCB does not inflate type-I error rate for association studies. But it should be noted that the conclusion made here is under the assumption that the extent of missing is same to case and control. Sometimes differential bias between case and control is unavoidable, i.e., for the different sourcing of samples. In this case, effects of MCB and genotyping errors could be more complicated. Clayton et al. [34] showed case-control differential bias and calling inaccuracies can lead to differential misclassification, and consequently, to increase false-positive rates. Plagnol et al. [35] from the same lab found case-control bias associated with missing data can increase the false-positive rate as well and recommended to use 'fuzzy' calls to deal with uncertain genotypes that would otherwise be labeled as missing.

## Conclusion

Missing calls in high-throughput genotyping has long been ignored in genetic studies. However, it had been illustrated that the problem of missing call bias does exist widely and sometimes seriously in prevalent high-throughput genotyping technologies. Missing call bias could lead to biased conclusions in subsequent analyses, including allele/genotype frequency estimation and association studies. The commonly used 'no-call' procedure does not always a best option for observations of borderline quality. Our results indicated that in most cases, the biased conclusion can be greatly reduced by increasing the call-rate at the cost of genotyping error rate. Therefore, the existing QC standards should be modified that the cut-off for call-rate and that for genotyping error rate should be properly coupled in GWA. A program called QC-Tradeoff is available online to suggest call or no-call the equivocal observations according to the case the user faced to minimize the power influences in association studies, and illustrate the acceptable QC standard in the corresponding case.

## Methods

### Regenotyping for no-calls

Two SNPs were selected for each platform (GeneChip 500 K, SNPstream, Illumina and TaqMan) from a large number of loci which were genotyped by the respective technology in our laboratory. The SNPs were selected using the following criteria: 1) the genotype calls were made using the software provided by the venders of the respective technology; 2) the call-rate at each locus is around or above the average call-rate from the same platform; 3) the minor allele frequency (MAF) of the observed data is above 0.15.

As for SNPstream, Illumina and TaqMan, their calling algorithms are based on various methods, including GetGenos/QCReview program for SNPstream, GeneCall software for Illumina and SDS software for TaqMan. But generally, a baseline is set to distinguish the background signals and the informative ones, and then clustering and calling procedures conduct in the informative ones. The samples that show signals above the baseline but cannot be called unequivocally were selected for further analysis in the respective genotyping platforms. As for GeneChip 500 K, genotype data was called by the software GTYPE. It introduces a dynamic model-based algorithm [36] to suit its properties that many different SNPs are to be examined in a few individuals. This algorithm is different from others, so instead, we collected all the missing samples for sequencing.

Overall, *rs6743724 *(call-rate: 96.5%) and *rs699512 *(98.6%) for SNPstream, *rs2277632 *(98.9%) and *rs1457043 *(98.9%) for Illumina, *rs10109984 *(95.8%) and *rs11226 *(96.6%) for TaqMan, *rs1192885 *(94.2%) and *rs6855202 *(95.1%) for GeneChip 500 K were selected. The genotypes for the missing data were generated by sequencing the DNA segment containing the polymorphism locus. In the 2 × 3 contingency table of genotype in the 'observed' data produced by the genotyping platforms (*Obs*.) and the 'missing' data produced by sequencing (*Seq*.), Fisher Exact Test was used to examine whether there is difference in the genotypic distribution between them (Missing Call Bias, MCB). The genotype-specific call-rate was calculated as well, $c_{i}=\frac{Obs_{i}}{Obs_{i}+Seq_{i}}$ for *AA*, *Aa *and *aa*, respectively.

### Models for MCB and genotyping errors

In the presence of no-calls, let $p_{G_{i}}$ denote the frequency of the genotypes *G*_*i *_(*i *= *AA, Aa, aa*) in the population and *c*_*i *_be the call-rate of the genotypes where 0 ≤ *c*_*i *_≤ 1. The observed genotype frequencies can be presented as $p_{G_{i}}^{obs}=\frac{c_{i}p_{G_{i}}}{\sum_{i}c_{i}p_{G_{i}}}$, where the overall call-rate is defined as $CallRate=\sum_{i}c_{i}p_{G_{i}}$. When *c*_*AA *_= *c*_*Aa *_= *c*_*aa*_, there is no bias in missing data, we called it Unbiased Missing (UBM). A violation to any of the equality would lead to MCB. In the presence of MCB, Scenario II is equivalent to Scenario III. To facilitate discussion, we assumed no-calls only happen in a specific genotype with 0 ≤ *c *≤ 1, i.e., in *AA *(Scenario I), in *Aa *(Scenario II & III), and in *aa *(Scenario IV).

On the other hand, an equivocal data point can be assigned to a genotype, which could lead to a genotyping error. Assume all the equivocal data points are called and let *conf *denote the proportion of genotypes that could be accurately called among these equivocal ones. The genotyping error rate is (1 - *conf*)(1 - *c*)*p*_*G*_, where *G *denotes the genotype of equivocal data points.

The observed genotype frequencies $p_{G_{i}}^{obs}$ in the models were listed in Table 3.

**Table 3:** The genotype frequencies in different scenarios in the presence of MCB or genotyping errors.

**Scenarios**	**Missing Call Bias**	**Genotyping Errors for No-Calls**
Scenario I	$$\begin{array}{l} p_{AA}^{obs}=cp_{AA}/(cp_{AA}+p_{Aa}+p_{aa})\\ p_{Aa}^{obs}=p_{Aa}/(cp_{AA}+p_{Aa}+p_{aa})\\ p_{aa}^{obs}=p_{aa}/(cp_{AA}+p_{Aa}+p_{aa}) \end{array}$$	$$\begin{array}{l} p_{AA}^{obs}=cp_{AA}+conf(1-c)p_{AA}\\ p_{Aa}^{obs}=p_{Aa}+(1-conf)(1-c)p_{AA}\\ p_{aa}^{obs}=p_{aa} \end{array}$$

Scenario II	$$\begin{array}{l} p_{AA}^{obs}=p_{AA}/(p_{AA}+cp_{Aa}+p_{aa})\\ p_{Aa}^{obs}=cp_{Aa}/(p_{AA}+cp_{Aa}+p_{aa})\\ p_{aa}^{obs}=p_{aa}/(p_{AA}+cp_{Aa}+p_{aa}) \end{array}$$	$$\begin{array}{l} p_{AA}^{obs}=p_{AA}+(1-conf)(1-c)p_{Aa}\\ p_{Aa}^{obs}=cp_{Aa}+conf(1-c)p_{Aa}\\ p_{aa}^{obs}=p_{aa} \end{array}$$

Scenario III	$$\begin{array}{l} p_{AA}^{obs}=p_{AA}/(p_{AA}+cp_{Aa}+p_{aa})\\ p_{Aa}^{obs}=cp_{Aa}/(p_{AA}+cp_{Aa}+p_{aa})\\ p_{aa}^{obs}=p_{aa}/(p_{AA}+cp_{Aa}+p_{aa}) \end{array}$$	$$\begin{array}{l} p_{AA}^{obs}=p_{AA}\\ p_{Aa}^{obs}=cp_{Aa}+conf(1-c)p_{Aa}\\ p_{aa}^{obs}=p_{aa}+(1-conf)(1-c)p_{Aa} \end{array}$$

Scenario IV	$$\begin{array}{l} p_{AA}^{obs}=p_{AA}/(p_{AA}+p_{Aa}+cp_{aa})\\ p_{Aa}^{obs}=p_{Aa}/(p_{AA}+p_{Aa}+cp_{aa})\\ p_{aa}^{obs}=cp_{aa}/(p_{AA}+p_{Aa}+cp_{aa}) \end{array}$$	$$\begin{array}{l} p_{AA}^{obs}=p_{AA}\\ p_{Aa}^{obs}=p_{Aa}+(1-conf)(1-c)p_{aa}\\ p_{aa}^{obs}=cp_{aa}+conf(1-c)p_{aa} \end{array}$$

### Effects on allele frequency estimation and type-I error rate for HWE

Let π denote the frequency for minor allele *A *in a population, where 0 ≤ π ≤ 0.5. Suppose HWE holds in the population, the genotype frequencies are *p*_*AA *_= π^2^, *p*_*Aa *_= 2π(1 - π) and *p*_*aa *_= (1 - π)^2^, respectively.

However, in the presence of MCB or genotyping errors, the estimation of π will be affected. Here, we present the results by the difference (π^*obs*^-π) to reflect the changes in the allele frequency estimation, where π^*obs *^is the estimated frequency of minor allele *A *in the existence of MCB or genotyping errors according to Table 3. In order to compare the effects introduced by MCB with that by genotyping errors, we try to find a *conf *threshold in the given *c *and MAF, where if the equivocal observations are called above the *conf *threshold, the changes of allele frequency estimation (|π^*obs*^-π|) caused by genotyping errors are smaller than those by MCB, and vice versa.

MCB and genotyping errors will also cause the violation of HWE. For biallelic locus, $\chi_{HWE}^{2}=N{\left[\frac{p_{AA}p_{aa}-{\left(p_{Aa}/2\right)}^{2}}{\pi (1-\pi )}\right]}^{2}$ can be used to test the departure from HWE, where N is the sample size. This statistic conforms to the *χ*^2 ^distribution with one degree of freedom (*df *= 1), when the null hypothesis holds [37]. However, in the presence of MCB or genotyping errors, it conforms to a non-central *χ*^2 ^distribution with *df *= 1 and the non-centrality parameter is $\lambda =N^{obs}{\left[\frac{p_{AA}^{obs}p_{aa}^{obs}-{\left(p_{Aa}^{obs}/2\right)}^{2}}{\pi^{obs}(1-\pi^{obs})}\right]}^{2}$, which can be calculated according to Table 3. The explicit derivation of the non-centrality parameter in the presence MCB or genotyping errors was illustrated in Supplemental Methods. Here, type-I error rate for HWE was calculated according to the non-central *χ*^2 ^distribution in the significant level of 0.05 and the sample size was assumed to 500, which was conducted in the package of R[38].

### Disease models and asymptotic power calculation

Let *h*_*AA*_, *h*_*Aa*_, *h*_*aa *_be the penetrances of a disease for the genotypes *AA*, *Aa *and *aa*, respectively, and D be the prevalence of the disease,

(1) $$D=\mathrm{Pr}(Disease)=\sum_{i}\mathrm{Pr}(G_{i})\mathrm{Pr}(Disease|G_{i})=p_{AA}h_{AA}+p_{Aa}h_{Aa}+p_{aa}h_{aa}.$$

Following the Bayesian formulation, the genotype frequencies in the case population are

(2) $$\mathrm{Pr}(G_{i}|Disease)=\frac{\mathrm{Pr}(G_{i})\mathrm{Pr}(Disease|G_{i})}{\mathrm{Pr}(Disease)}=\frac{p_{G_{i}}h_{G_{i}}}{D}.$$

Similarly, the genotype frequencies in the control population are,

(3) $$\mathrm{Pr}(G_{i}|Normal)=\frac{\mathrm{Pr}(G_{i})\mathrm{Pr}(Normal|G_{i})}{\mathrm{Pr}(Normal)}=\frac{\mathrm{Pr}(G_{i})[1-\mathrm{Pr}(Disease|G_{i})]}{1-\mathrm{Pr}(Disease)}=\frac{p_{G_{i}}(1-h_{G_{i}})}{1-D}.$$

The aim of case-control association studies is to search for disease-susceptible polymorphisms by testing whether allele/genotype frequencies of the case and control differ significantly. Allelic *χ*^2 ^test and genotypic *χ*^2 ^test are the most two widely used tests. Here we examined the influences affected by MCB, UBM or genotyping errors on the power in association studies. The genotype frequencies in case and control can be calculated respectively according to Table 3 and formula (1)~(3). The power was calculated using a non-central *χ*^2 ^distribution following Gordon et al. [23]. The power calculation was conducted in the package of R. In order to the comparison of power affected by MCB and that by genotyping errors with different levels, we tend to find a *conf *threshold, where if the equivocal observations are called above the *conf *threshold, the power loss caused by genotyping errors are smaller than that by MCB, and vice versa.

Here, the same extent of MCB, UBM or genotyping errors was assumed for both case and control. Various modes of inheritance relationships were considered, including dominant, recessive, overdominant, additive and multiplicative relationship (Table 2). The power in these disease models can be 80% in the significant of 0.05 using genotypic *χ*^2 ^test, when MAF is 0.25 and the sample size of case and control is 500 respectively.

## Abbreviations

MCB: missing call bias; UBM: unbiased missing; SNP: single nucleotide polymorphism; QC: quality control; HWE: Hardy-Weinberg Equilibrium.

## Authors' contributions

WF, YiW and LJ conceived and designed the study. WF, YingW, RuiL and RongL carried out sequencing and genotyping experiments. WF analyzed the data and drafted the manuscript. LJ revised the manuscript. All authors read and approved the final manuscript.

## Supplementary Material

Additional File 1 **The power comparison among the null (printed in green), UBM (printed in yellow) and MCB (printed in red) under various disease models (dominant, recessive, overdominant and additive relationship) in the significant level of 0.05 when allelic *χ*^2 ^test was used.** The figures correspond to Scenario I, Scenario II & III and Scenario IV from the left to right. Click here for file

Additional File 2 **The power comparison among the null (printed in green), UBM (printed in yellow) and MCB (printed in red) under various disease models (dominant, recessive, overdominant and additive relationship) in the significant level of 0.05 when genotypic *χ*^2 ^test was used.** The figures correspond to Scenario I, Scenario II & III and Scenario IV from the left to right. Click here for file

Additional File 3 **Effects of genotyping errors on allele frequency estimation and on power of association studies under various disease models in the significant level of 0.05 when allelic *χ*^2 ^test (printed in green) and genotypic *χ*^2 ^test (printed in yellow) were used.** When *conf *= 1, the changes of MAF estimation is 0 and the power corresponds to that in the null. The figures correspond to Scenario I, Scenario II, Scenario III and Scenario IV from the left to right. Click here for file

Additional File 4 **The power comparison for association studies between MCB and genotyping errors under various disease models (dominant, recessive, overdominant, additive relationship) when allelic *χ*^2 ^test was used.A) **illustrates the overall call-rate for the loci with different MAFs in the presence of MCB (*c *= 0.8). **B) **illustrates the threshold of *conf *by a solid line. If the equivocal observations can be called accurately in a confidence above the *conf *threshold, it prefers to call those equivocal ones at the cost of genotyping errors to minimize the power loss (grey area above the line). Otherwise, 'no-call' procedure is beneficial, which results in MCB (area below the line). **C) **illustrates the genotyping error rate, when the equivocal observations are called in the *conf *threshold mentioned above. The figures correspond to Scenario I, Scenario II, Scenario III and Scenario IV from the left to right. Click here for file

Additional File 5 **The power comparison for association studies between MCB and genotyping errors under various disease models (dominant, recessive, overdominant, additive relationship) when genotypic *χ*^2 ^test was used.A) **illustrates the overall call-rate for the loci with different MAFs in the presence of MCB (*c *= 0.8). **B) **illustrates the threshold of *conf *by a solid line. If the equivocal observations can be called accurately in a confidence above the *conf *threshold, it prefers to call those equivocal ones at the cost of genotyping errors to minimize the power loss (grey area above the line). Otherwise, 'no-call' procedure is beneficial, which results in MCB (area below the line). **C) **illustrates the genotyping error rate, when the equivocal observations are called in the *conf *threshold mentioned above. The figures correspond to Scenario I, Scenario II, Scenario III and Scenario IV from the left to right. Click here for file

Additional File 6 **Joint effects of MCB and genotyping errors on association studies under various disease models (dominant, recessive, overdominant, additive relationship).A) **illustrates the overall call-rate for the loci with different values of *α *in the joint models of MCB and genotyping errors (MAF = 0.25, *c *= 0.8, *conf *= 0.0, 0.25, 0.5, 0.75 and 1.0). **B) **illustrates the genotyping error rate with different values of *α *in the corresponding joint models. **C) **illustrates the power based on allelic *χ*^2 ^test with different values of *α*. **D) **illustrates the power based on genotypic *χ*^2 ^test with different values of *α*. The figures correspond to Scenario I, Scenario II, Scenario III and Scenario IV from the left to right. Click here for file

Additional File 7 **The non-centrality parameter of HWE test in the presence of MCB or genotyping errors; the joint model of MCB and genotyping errors.** Click here for file
