# Supplementary material for: Missing call bias in high-throughput genotyping
Source: BMC Genomics. 2009 Mar 13;10:106. doi: 10.1186/1471-2164-10-106 (PMC2670840; doi:10.1186/1471-2164-10-106)

Dominant Disease Model

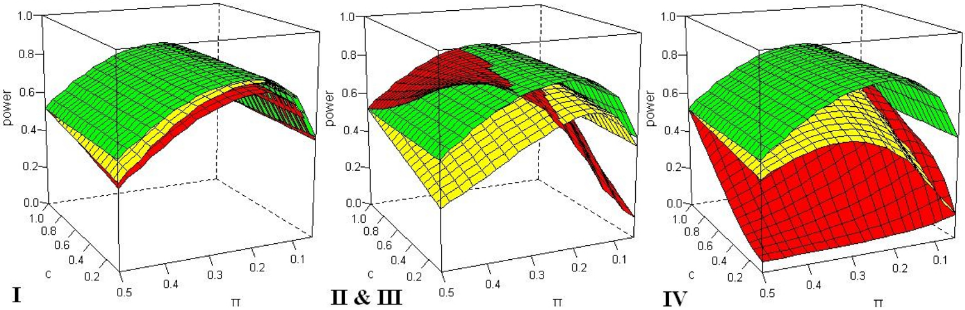

Recessive Disease Model

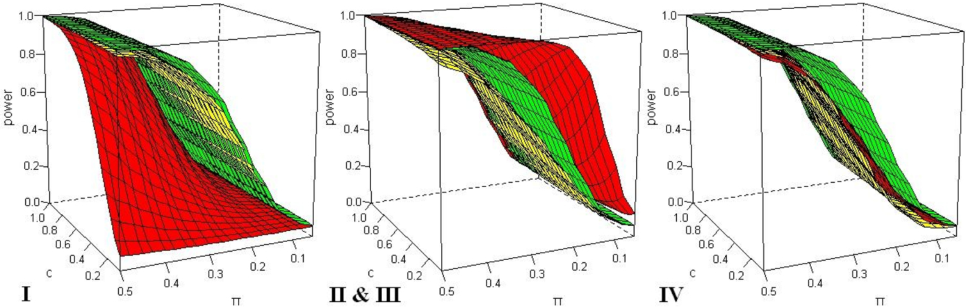

Overdominant Disease Model

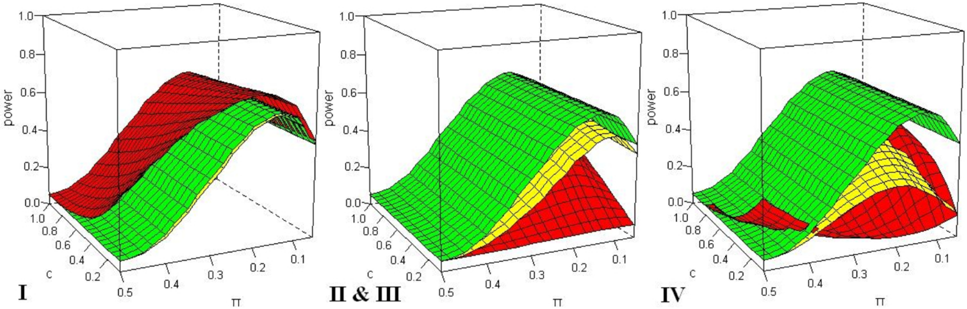

Additive Disease Model

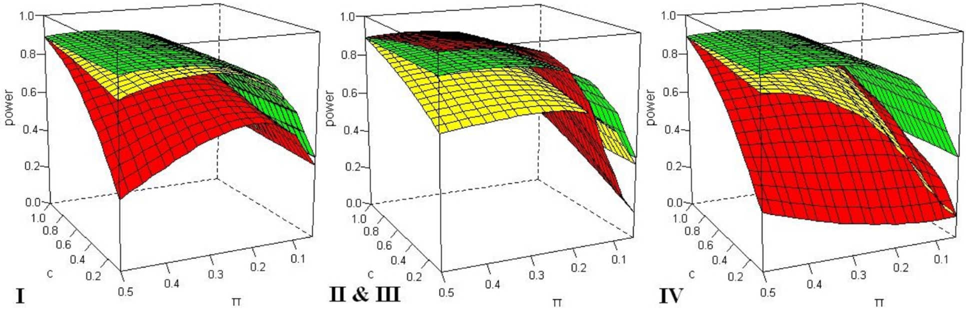

Supplement: Additional File 1 — The power comparison among the null (printed in green), UBM (printed in yellow) and MCB (printed in red) under various disease models (dominant, recessive, overdominant and additive relationship) in the significant level of 0.05 when allelic χ2 test was used. The figures correspond to Scenario I, Scenario II & III and Scenario IV from the left to right. [file 1471-2164-10-106-S1.pdf]
