# Supplementary material for: Missing call bias in high-throughput genotyping
Source: BMC Genomics. 2009 Mar 13;10:106. doi: 10.1186/1471-2164-10-106 (PMC2670840; doi:10.1186/1471-2164-10-106)

Changes of Allele Frequency Estimation

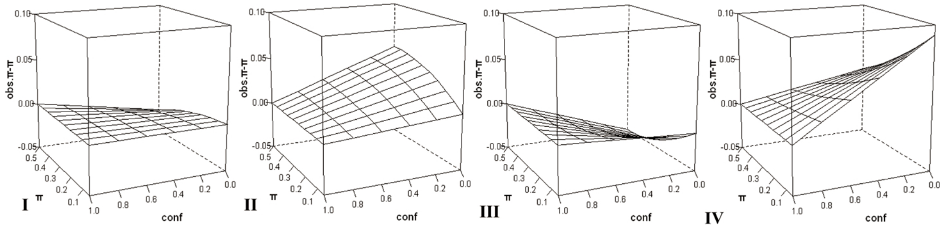

Multiplicative Disease Model

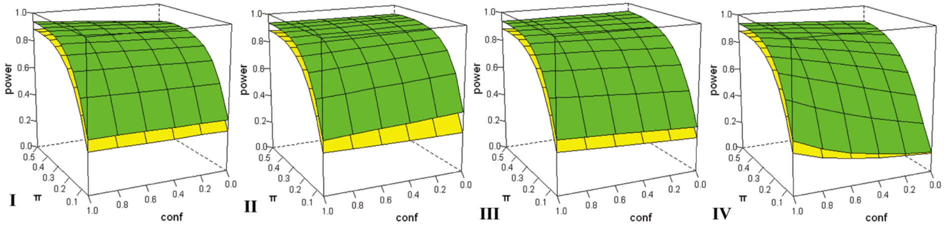

Dominant Disease Model

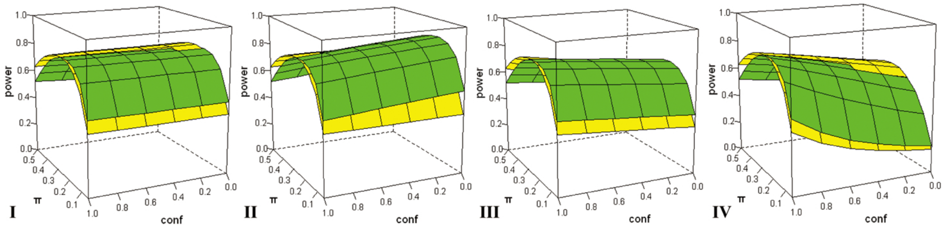

Recessive Disease Model

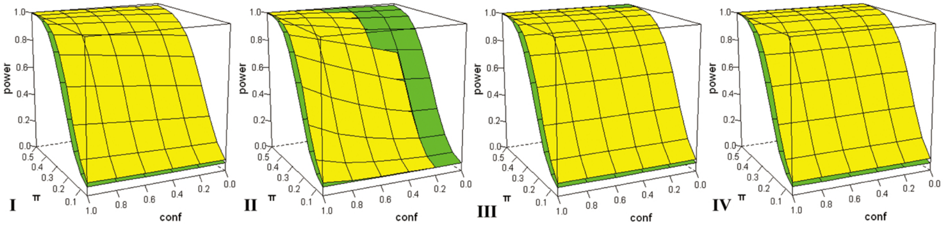

Overdominant Disease Model

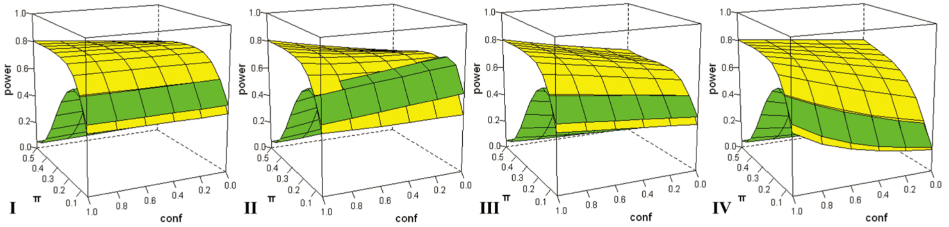

Additive Disease Model

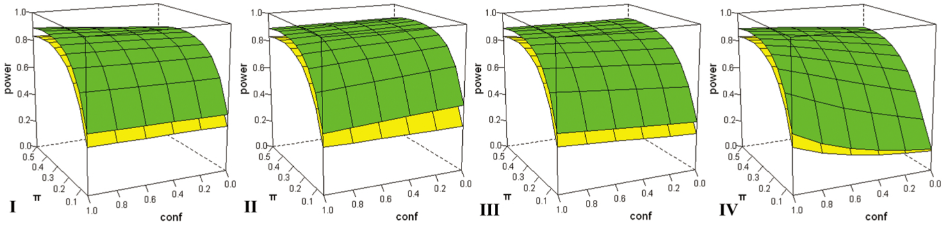

Supplement: Additional File 3 — Effects of genotyping errors on allele frequency estimation and on power of association studies under various disease models in the significant level of 0.05 when allelic χ2 test (printed in green) and genotypic χ2 test (printed in yellow) were used. When conf = 1, the changes of MAF estimation is 0 and the power corresponds to that in the null. The figures correspond to Scenario I, Scenario II, Scenario III and Scenario IV from the left to right. [file 1471-2164-10-106-S3.pdf]
