# Supplementary material for: Missing call bias in high-throughput genotyping
Source: BMC Genomics. 2009 Mar 13;10:106. doi: 10.1186/1471-2164-10-106 (PMC2670840; doi:10.1186/1471-2164-10-106)

Dominant Disease Model

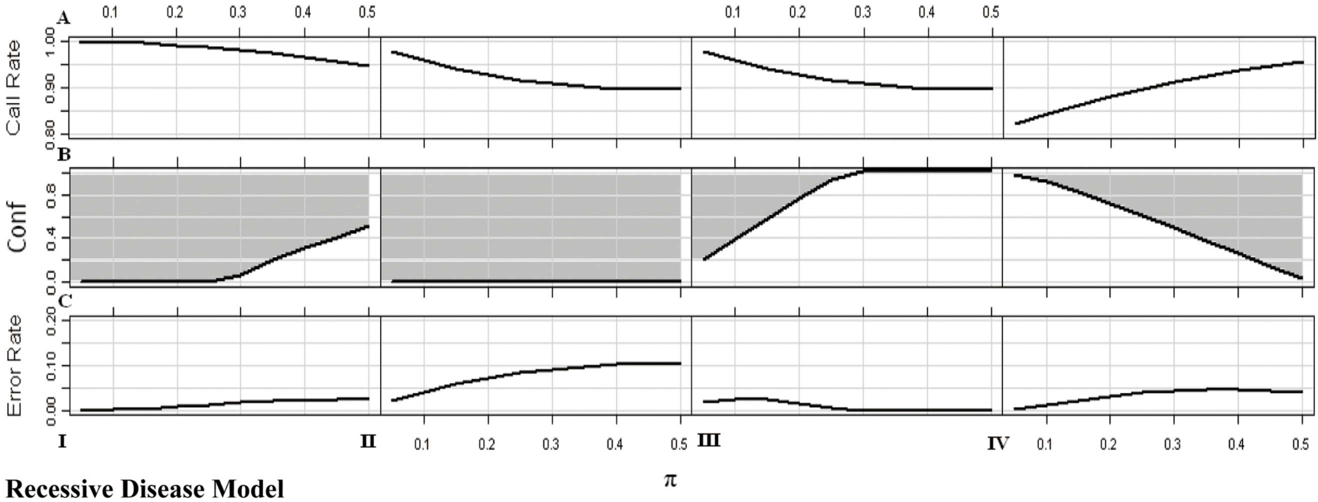

Recessive Disease Model

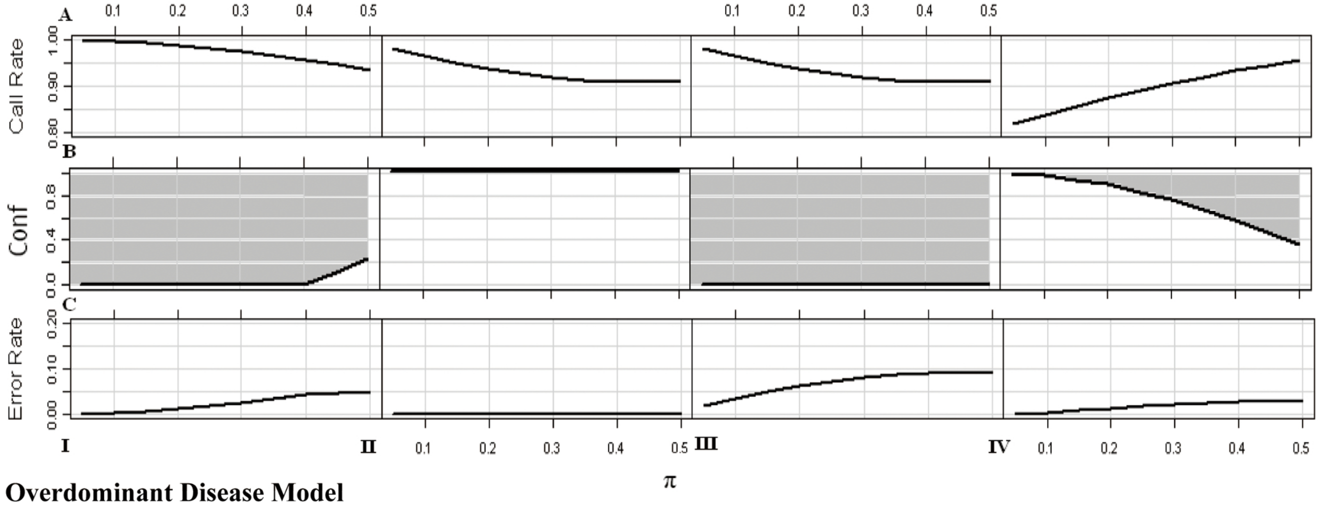

Overdominant Disease Model

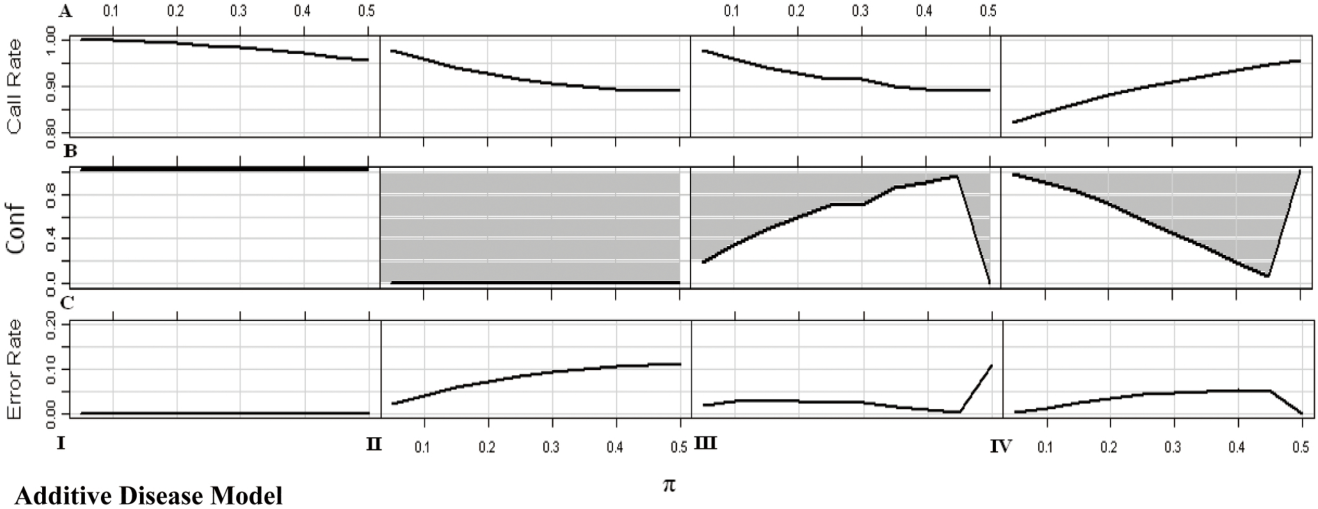

Additive Disease Model

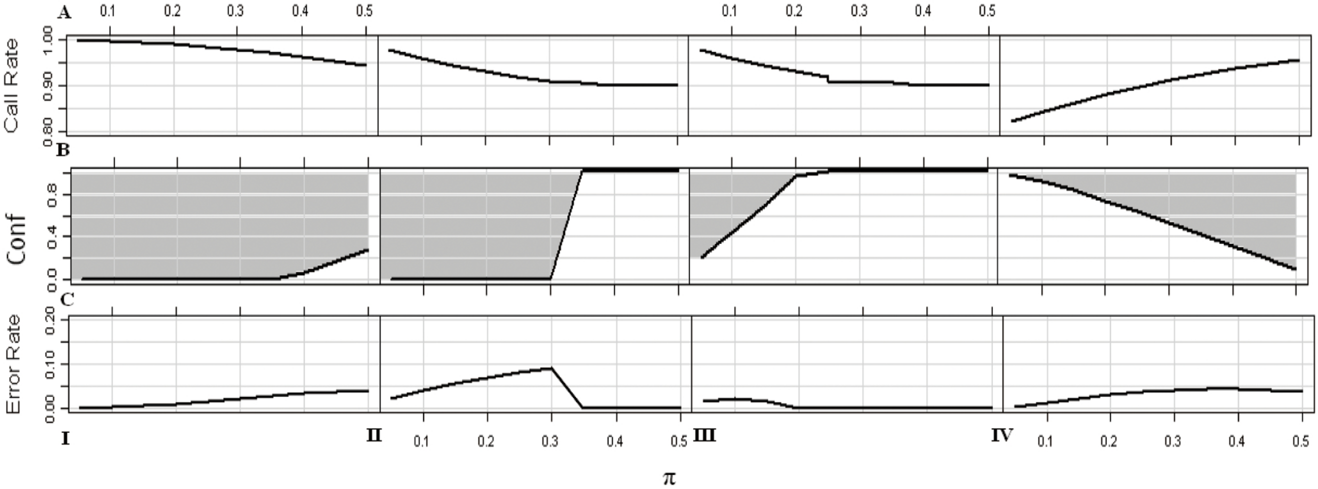

Supplement: Additional File 4 — The power comparison for association studies between MCB and genotyping errors under various disease models (dominant, recessive, overdominant, additive relationship) when allelic χ2 test was used.A) illustrates the overall call-rate for the loci with different MAFs in the presence of MCB (c = 0.8). B) illustrates the threshold of conf by a solid line. If the equivocal observations can be called accurately in a confidence above the conf threshold, it prefers to call those equivocal ones at the cost of genotyping errors to minimize the power loss (grey area above the line). Otherwise, 'no-call' procedure is beneficial, which results in MCB (area below the line). C) illustrates the genotyping error rate, when the equivocal observations are called in the conf threshold mentioned above. The figures correspond to Scenario I, Scenario II, Scenario III and Scenario IV from the left to right. [file 1471-2164-10-106-S4.pdf]
