# Supplementary material for: Missing call bias in high-throughput genotyping
Source: BMC Genomics. 2009 Mar 13;10:106. doi: 10.1186/1471-2164-10-106 (PMC2670840; doi:10.1186/1471-2164-10-106)
Supplement: Additional File 6 — Joint effects of MCB and genotyping errors on association studies under various disease models (dominant, recessive, overdominant, additive relationship).A) illustrates the overall call-rate for the loci with different values of α in the joint models of MCB and genotyping errors (MAF = 0.25, c = 0.8, conf = 0.0, 0.25, 0.5, 0.75 and 1.0). B) illustrates the genotyping error rate with different values of α in the corresponding joint models. C) illustrates the power based on allelic χ2 test with different values of α. D) illustrates the power based on genotypic χ2 test with different values of α. The figures correspond to Scenario I, Scenario II, Scenario III and Scenario IV from the left to right. [file 1471-2164-10-106-S6.pdf]

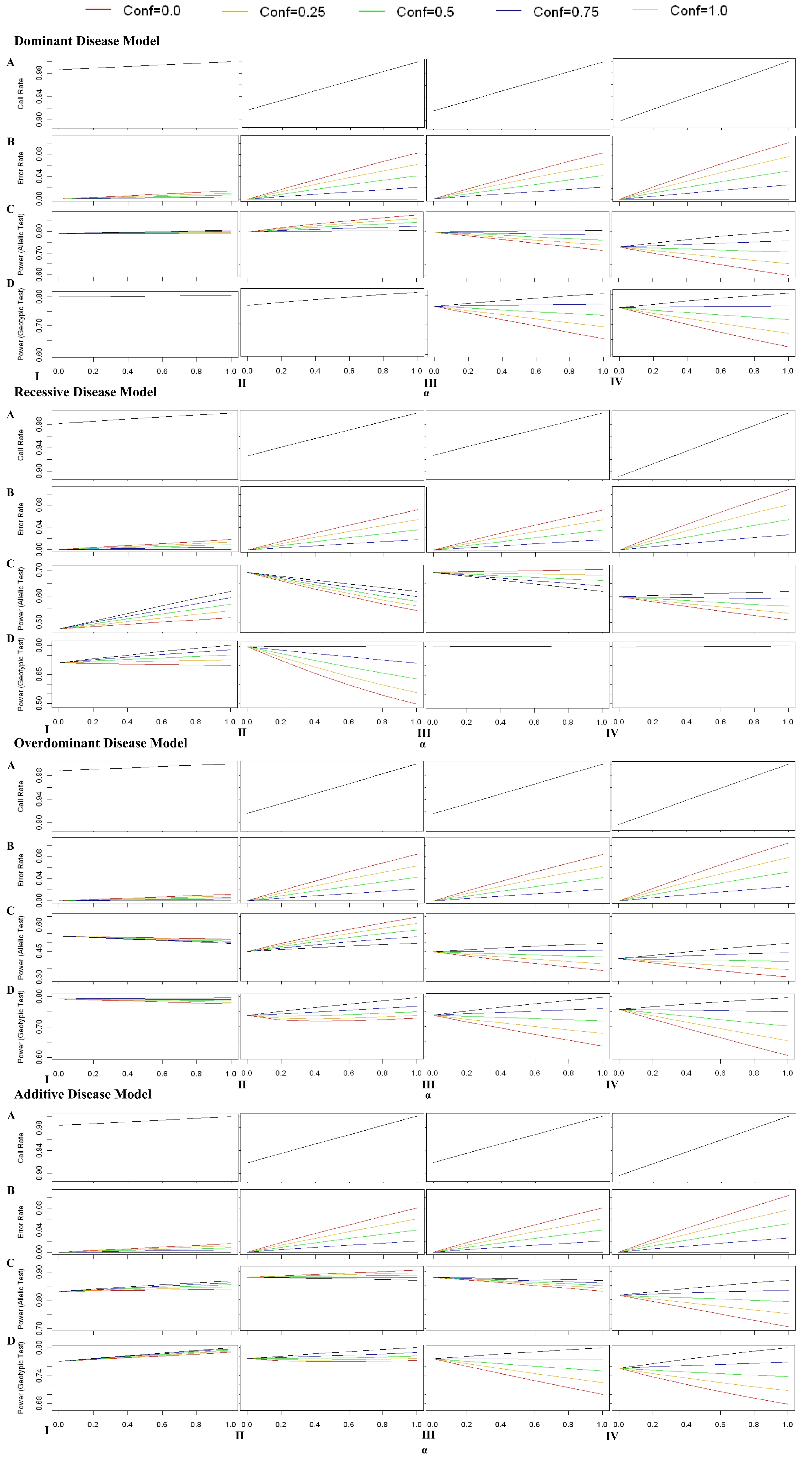
Conf=0.0
Conf=0.25
Conf=0.5
Conf=0.75
Conf=1.0
Dominant Disease Model
Recessive Disease Model
Overdominant Disease Model
Additive Disease Model
A
B
C
D
Call Rate
Error Rate
Power (Allelic Test)
Power (Geotypic Test)
I
II
III
IV
α
