# Supplementary material for: Missing call bias in high-throughput genotyping
Source: BMC Genomics. 2009 Mar 13;10:106. doi: 10.1186/1471-2164-10-106 (PMC2670840; doi:10.1186/1471-2164-10-106)
Supplement: Additional File 7 — The non-centrality parameter of HWE test in the presence of MCB or genotyping errors; the joint model of MCB and genotyping errors. [file 1471-2164-10-106-S7.doc]

**Supplemental Methods**

*The non-centrality parameter of HWE test in the presence of MCB or genotyping errors*

The departure form HWE caused by MCB or genotyping error could be measured by the type-I error rate of a non-central *χ^2^* distribution with 1 degree of freedom. The non-centrality parameter is . An explicit derivation of the non-centrality parameter in different scenarios was given as follows.

1. In models for MCB,
2. For Scenario I,
3. For Scenario II & III,
4. For Scenario IV,
5. In models for genotyping errors,
6. For Scenario I,
7. For Scenario II,
8. For Scenario III,
9. For Scenario IV,

*The joint model of MCB and genotyping errors*

According to the model of MCB, the proportion of equivocal observations for a specific genotype G is 100×(1-*c*) %. We assumed only (100×α) % of equivocal observations were called with accuracy denoted still by *conf*. In the joint model of MCB and genotyping errors, the observed genotype frequencies are,

In Scenario I,

In Scenario II,

In Scenario III,

In Scenario IV,

.

It could be inferred that when α=0, it is the model for MCB; and when α=1, it is the model for genotyping errors.
